# Willoughby Medical Institution

**Published:** 1846-12

**Authors:** 


ARTICLE XVIII.

### WILLOUGHBY MEDICAL INSTITUTION.

The Faculty of Willoughby Medical School are now giving, as we are informed upon good authority, their last course of lectures.

To a large number of the physicians in this region, it may appear surprising that a school, which numbered upwards of 160 students in the class of last season,—which has so respectable and active a faculty,—which has its graduates scattered through the west, should suspend operations. To those, however, acquainted with the situation of the town of Willoughby, and the great disadvantages it labored under, it has long been obvious that a school at that place could only be supported by the excessive and constant efforts of its faculty and friends. The reason is obvious: it is the proximity of the place to Cleveland, where the advantages afforded to students, are, or will be, every way superior. Cleveland is the natural centre of business for Northern Ohio, and individual efforts will not long contend against natural and constantly operating causes in any department of human affairs. Another cause has, we doubt not, contributed to discourage the faculty at that place; it is the almost unlimited adoption of the credit system, whereby, with the gratification of lecturing to a large class, professors are forced to submit to the inconveniences of empty pockets.

Whatever may be the causes, we congratulate the profession in this region on the result which is the suspension of the school at Willoughby, and its transfer, with some modifications of faculty, of which we are not fully informed, to Columbus, Ohio. In this new region, and the adjoining part of Indiana, there are a large number of students and practitioners, not graduates, who will be benefited by the transfer, and we hope now to see the faculty of the Cleveland Medical College restore the price of their tickets to the ordinary standard of the northern states, a course dictated by their own interests, as well as those of the profession at large.

